# Hemophagocytic Lymphohistiocytosis in Visceral Leishmaniasis: A Rare yet Treatable Complication

**DOI:** 10.7759/cureus.70104

**Published:** 2024-09-24

**Authors:** Anupam Varshney, Ranjan Solanki, David Manna, Efren Rodriguez, Vijay K Bindra

**Affiliations:** 1 Department of Pathology, Muzaffarnagar Medical College and Hospital, Muzaffarnagar, IND; 2 Department of Basic Biomedical Sciences, Touro College of Osteopathic Medicine, Middletown, USA; 3 Department of Medicine, Jaswant Rai Speciality Hospital, Meerut, IND

**Keywords:** amastigote forms, bone marrow aspirate, hemophagocytic lymphohistiocytosis, leishmania, pancytopenia

## Abstract

Hemophagocytic lymphohistiocytosis (HLH) is a rare debilitating condition that can be triggered by an infectious cause, often linked to the Epstein-Barr virus (EBV). In this case, we evaluated a patient with pancytopenia. The bone marrow aspiration revealed the presence of amastigotes and active hemophagocytosis, indicating that the HLH was induced by a* Leishmania* infection. The patient was treated with lyophilized amphotericin B followed by miltefosine, which effectively resolved the infection and HLH. This case report underscores the presentation and findings of *Leishmania*-induced HLH, as well as the successful treatment approach that led to improved patient outcomes.

## Introduction

Hemophagocytic lymphohistiocytosis (HLH) is a disease involving the dysregulated activity of macrophages, natural killer (NK) cells, and cytotoxic T lymphocytes, resulting in hypercytokinemia and immune-mediated injury to various organ systems [1]. HLH can occur due to genetic disorders or can be triggered by an inflammatory insult, usually seen secondary to an Epstein-Barr virus (EBV) infection, which is a major cause; other common viral etiologies include cytomegalovirus, herpes simplex virus, varicella zoster, measles, human herpesvirus 8 (HHV8), H1N1 influenza, and HIV [2-4]. Many tropical infections, such as malaria, scrub typhus, dengue, and leishmaniasis, have been reported to trigger HLH [5]. In this case, we investigate a rare incidence of HLH secondary to a* Leishmania* infection. HLH induced by leishmaniasis accounts for 0.77-2.1% of HLH cases, and the first recorded case of HLH in North America secondary to leishmaniasis was published in 2013 [6,7].

## Case presentation

**Figure 1:**
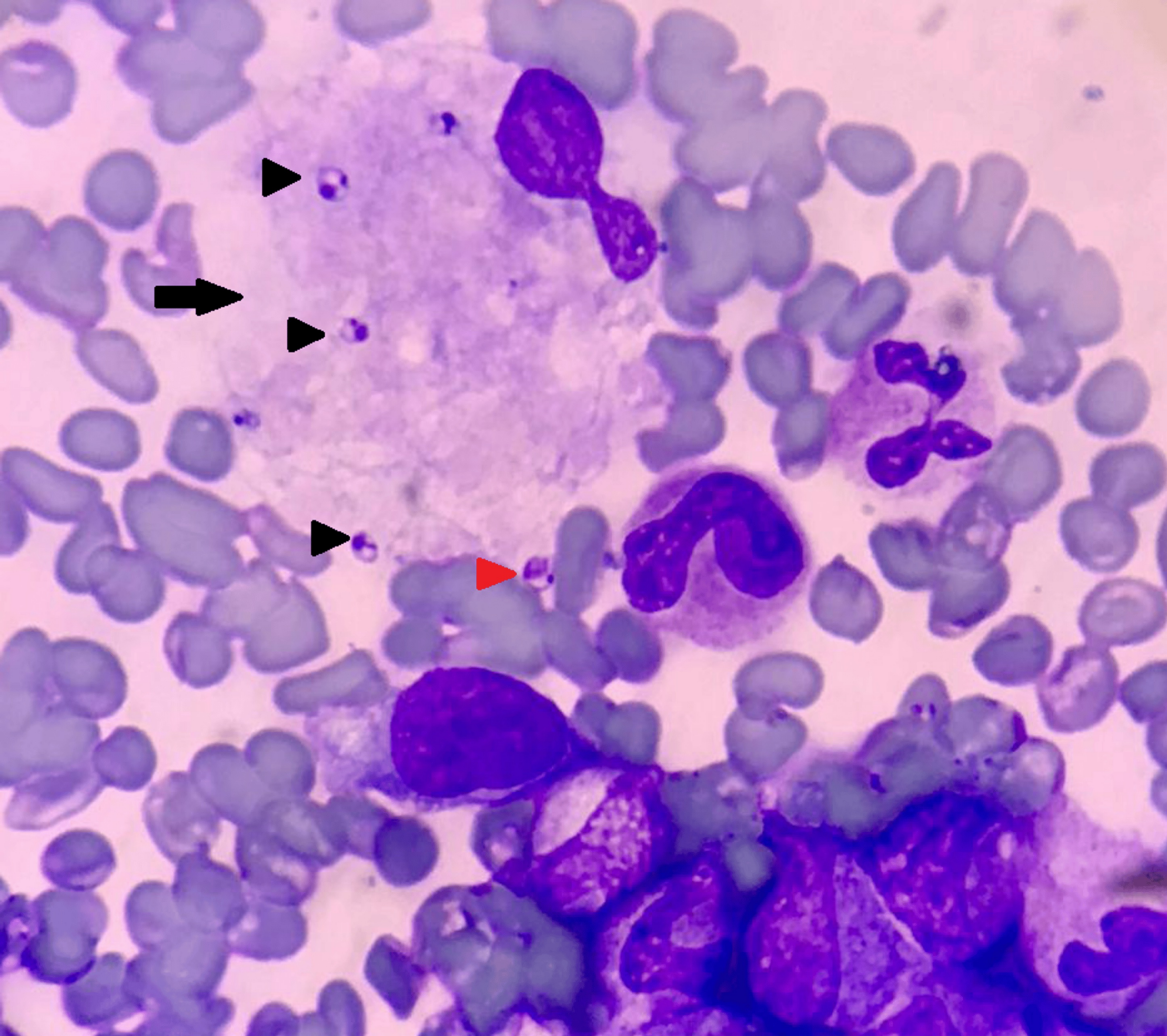
Bone marrow aspirate (Wright-Giemsa stain, 1000×) A macrophage (arrow) containing numerous amastigote forms (LD bodies) of *Leishmania* (black arrowheads). One extracellular LD body is also present (red arrowhead) along with numerous marrow cells and RBCs. LD: *Leishmania* *donovani*; RBCs: red blood cells

**Figure 2:**
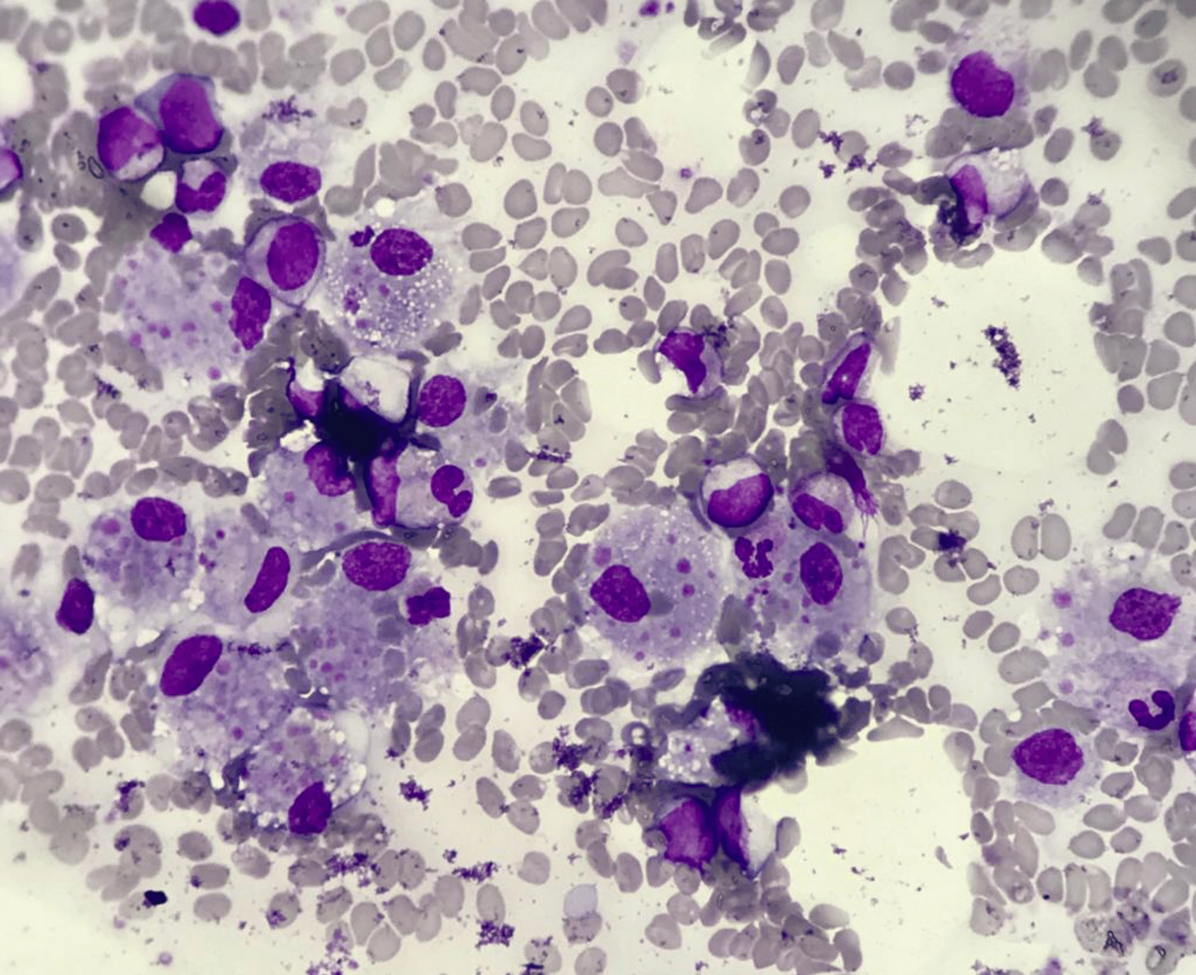
Bone marrow aspirate (Wright-Giemsa stain, 400×) Active hemophagocytosis revealing numerous macrophages with phagocytosed RBCs, platelets, and occasional WBC. RBCs: red blood cells; WBC: white blood cell

A 27-year-old woman presented to the hospital with a fever that had persisted for one week. She had been taking over-the-counter medications which did not alleviate her symptoms. Over the past three days, her fever worsened, and she developed petechiae throughout her body, along with abdominal pain. On physical examination, she was febrile (103.1°F) and had pallor, petechiae, a palpable spleen over 5 cm below the costal margin, and mild hepatomegaly. Based on clinical presentation, infections such as malaria and dengue, as well as acute leukemia with secondary infections, were considered as the probable differential diagnoses, and laboratory tests were requested. Laboratory work revealed a hemoglobin level of 7.2 (12-16) g/dL, a white blood cell (WBC) count of 2,700 (4500-11,000) cells/mm^3^, and an undetectable platelet count on the automated cell counter. A digital microscopy was performed to assess the platelet count for any artifacts, but the examination of the peripheral smear confirmed a severe degree of thrombocytopenia, which was reflected in a clinical manifestation as petechiae in the patient [8]. However, the patient's prothrombin time and partial prothrombin time were within the normal range. The serum ferritin level was assessed and found to be 3750 (10-120) ng/mL. Alanine aminotransferase (ALT) level was 129 (10-40) U/L, aspartate aminotransferase (AST) was 306 (12-38) U/L, and alkaline phosphatase was 138 (25-100) U/L (Table 1). The tests for malaria and dengue were negative, and no leukemic cells were found in the peripheral blood. The patient was admitted to the intensive care unit and was stabilized. Platelet units were infused to effectively treat thrombocytopenia, and the platelet count was closely monitored. Considering the lab findings, a bone marrow aspiration was performed to determine the cause of the patient's symptoms. This bone marrow aspiration revealed trilineage hematopoiesis with hyperplastic changes along with characteristic findings revealing histiocytes with phagocytosed red blood cells, WBC, and platelets, a characteristic microscopy finding reflecting HLH (Figure 1). Active hemophagocytosis was frequent, counting about 1-2 histiocytes with ingested blood cells per high-power field, and in some fields, the count was even more (Figure 2). Further evaluation of the bone marrow revealed numerous amastigote forms of *Leishmania* present intracellularly, particularly within macrophages, and extracellularly as well. No findings suggesting malignancies or any other infiltrating disorder capable of disrupting hematopoiesis were found. The patient's chest X-ray was normal, while her abdominal ultrasonography confirmed hepatosplenomegaly and revealed lymphadenopathy without any fluid collection. The patient's medical history did not suggest any primary/genetic disease manifesting as HLH. Furthermore, a lipid profile was assessed, and the results showed hypertriglyceridemia of 172.3 (less than 150) mg/dL (Table 1). The addition of all the relevant test results yielded an HScore of 254 for reactive hemophagocytic syndrome [9]. Based on all the evidence on hand, the diagnosis of HLH secondary to leishmaniasis was made by meeting ≥5 of the criteria set by the HLH-2004 trial, which includes fever, splenomegaly, cytopenia, hypertriglyceridemia and/or hypofibrinogenemia, hemophagocytosis phenomena, low NK-cell activity, hyperferritinemia, and elevated soluble interleukin 2 receptor (sCD25) [10]. A treatment plan was devised, and the patient was administered lyophilized amphotericin B followed by oral miltefosine. The patient responded well to the treatment, and her symptoms were resolved by the eighth day of admission. After exhibiting positive progress in her lab tests, particularly with a platelet count of 141,000 cells/mm^3^ and a negative *Leishmania* PCR test, the patient was monitored and subsequently discharged on the 11th day. During the initial follow-up visit three weeks after discharge, her lab parameters were within normal range. 

**Table 1:** Laboratory investigations CBC: complete blood count; MCV: mean corpuscular volume; MCH: mean corpuscular hemoglobin; MCHC: mean corpuscular hemoglobin concentration; INR: international normalized ratio; KFT: kidney function test; BUN: blood urea nitrogen; LFT: liver function test; AST: aspartate aminotransferase; ALT: alanine aminotransferase; HDL: high-density lipoprotein; LDL: low-density lipoprotein; NS1: non-structural protein 1

Test	Result	Reference range
CBC		
Hemoglobin	7.2 g/dL	12-16 g/dL
White blood cell count	2,700 cells/mm^3^	4,500-11,000 cells/mm^3^
MCV	98.1 µm^3^	80-100 µm^3^
MCH	32.7 pg/cell	25-35 pg/cell
MCHC	33.3%	31-36%
Platelet count	Not detectable	150,000-400,000 cells/mm^3^
Erythrocyte count	2.2 million/mm^3^	3.5-5.5 million/mm^3^
Coagulation profile		
Bleeding time	More than 15 minutes	1-9 minutes
Prothrombin time	13 seconds	11-15 seconds
INR	1.1	0.8-1.1
Activated partial thromboplastin time	32 seconds	25-40 seconds
KFT		
BUN	14.2 mg/dL	7-18 mg/dL
Creatinine	0.9 mg/dL	0.6-1.2 mg/dL
LFT		
AST	306 U/L	12-38 U/L
ALT	129 U/L	10-40 U/L
Alkaline phosphatase	138 U/L	25-100 U/L
Serum bilirubin, total	0.7 mg/dL	0.1-1 mg/dL
Serum bilirubin, direct	0.3 mg/dL	0-0.3 mg/dL
Serum proteins, total	5.9 g/dL	6-7.8 g/dL
Albumin	3.2 g/dL	3.5-5.5 g/dL
Globulin	2.7 g/dL	2.3-3.5 g/dL
Lipid profile		
Serum cholesterol, total	168.6 mg/dL	<200 mg/dL
HDL	47.5 mg/dL	40-60 mg/dL
LDL	86.6 mg/dL	<160 mg/dL
Triglycerides	172.3 mg/dL	<150 mg/dL
Ferritin and serologic tests		
Serum ferritin	3750 ng/mL	10-120 ng/mL
Malaria antigen test	Negative	Negative
Dengue NS1 and antibody test	Negative	Negative

## Discussion

HLH represents a perilous systemic hyperinflammatory syndrome marked by fever, elevated ferritin levels, other indicators of systemic inflammation, diminished blood cell counts, disseminated intravascular coagulopathy, hepatitis, and central nervous system (CNS) inflammation. This condition poses a significant risk for multiple organ dysfunction and shock and frequently culminates in fatality [11,12]. The HLH features severe cytopenias due to uncontrolled hemophagocytosis. Other laboratory signs and clinical symptoms result from disordered immune regulation and cytokine storm. On a broader scale, HLH is divided into HLH syndrome, HLH disease, and HLH disease mimics; the term HLH syndrome refers to a pathologic immune activation that is often associated with an underlying genetic abnormality of lymphocyte cytotoxicity, whereas HLH disease may be associated with specific genetic and/or environmental causes [13]. The HLH disease mimic resembles HLH syndrome but is secondary to other conditions.

The immunopathological state observed in HLH necessitates a methodical assessment of etiological factors and expeditious intervention. It can manifest in diverse contexts, complicating a broad spectrum of diseases, often with uncertain etiologies [12]. After a diagnosis of HLH, it is important to carefully weigh the potential benefits and risks of immunosuppressive therapy. Treatment may be customized based on the underlying cause. In the acute phase, it's crucial to balance the need for a thorough diagnostic process (including evaluating for lymphoma and infection) with the requirement for prompt treatment [14]. However, recognizing the underlying specific etiology, which can be achieved in some cases, and devising an appropriate management plan can change the overall outcome and eliminate the associated complications. Recognizing the clinical and laboratory features of HLH, identifying underlying contributors, initiating appropriate (empiric, targeted, and prophylactic) treatments, and monitoring for response, progression, and complications are essential to prevent fatal outcomes [12].

**Figure 3:**
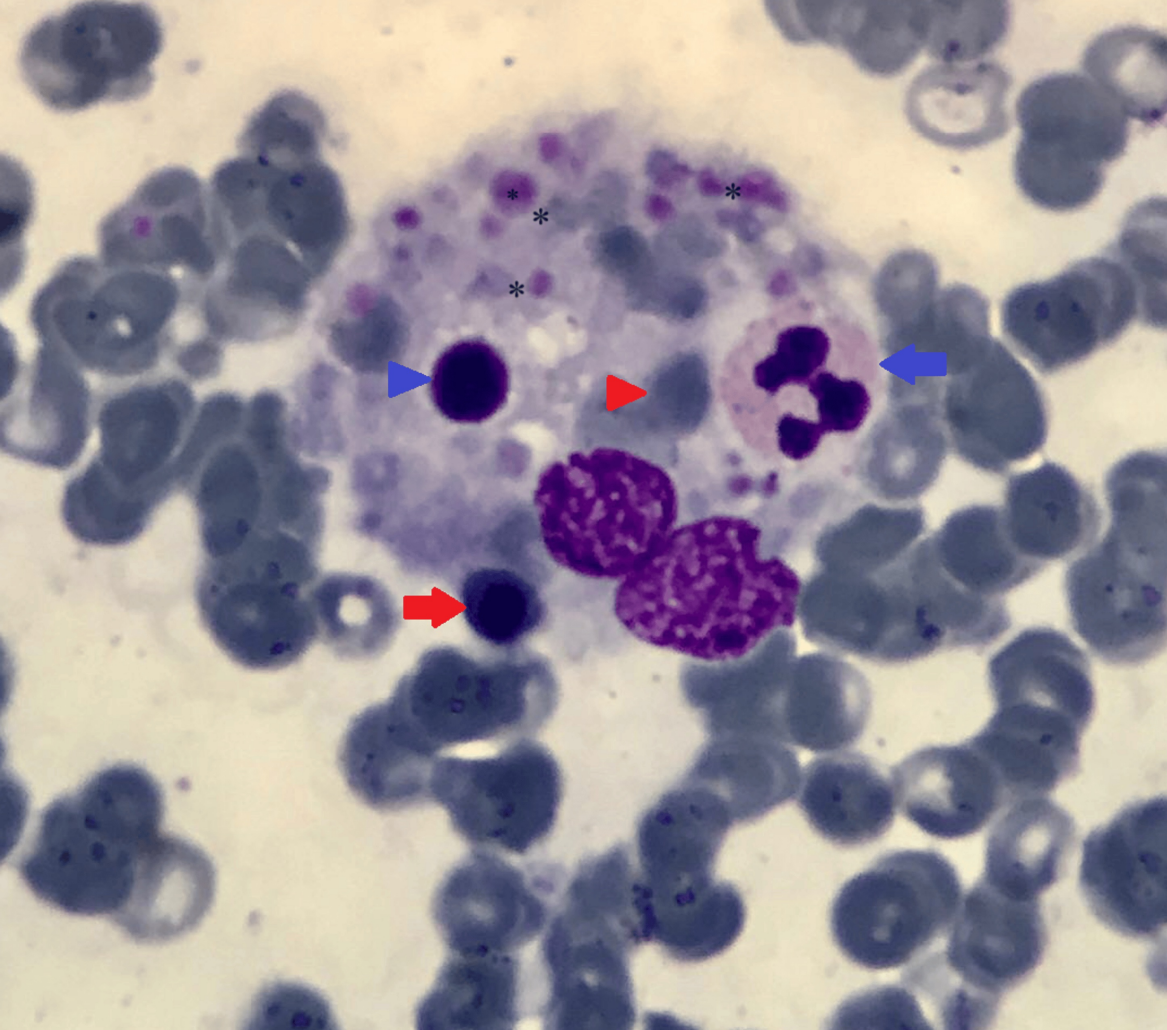
Bone marrow aspirate (Wright-Giemsa stain, 1000×) Active hemophagocytosis: a macrophage with phagocytosed RBCs (red arrowhead), late normoblast (red arrow), platelets (asterisk), lymphocyte (blue arrowhead), and neutrophil (blue arrow). RBCs: red blood cells

In this case, we confirmed the underlying etiology as a *Leishmania* infection, which helped manage the patient. Leishmaniasis is a parasitic disease in parts of the tropics, subtropics, and Southern Europe. It is classified as a neglected tropical disease (NTD). From 1990 to 2017, there was a significant decrease in age-standardized disability-adjusted life year (DALY) rates for visceral leishmaniasis (97.8% (97-99.2%)) [15]. However, the unusual occurrence of HLH significantly impacts prognosis [16]. The bite of phlebotomine sandflies spreads *Leishmania* parasites, which are obligate intracellular protozoa of the genus *Leishmania*; infections in humans are caused by roughly 21 of the 30 species that infect mammals. These include the *Leishmania donovani* (LD) *complex* with three species (*L. donovani*, *L. infantum*, and *L. chagasi*), the *L. mexicana complex (L. tropica*,* L. major*,and* L. aethiopica*), and the subgenus *Viannia*. The different species are morphologically indistinguishable, but isoenzyme analysis, molecular methods, or monoclonal antibodies can differentiate them [17]. There are three main types of leishmaniasis: (1) visceral, often known as kala-azar and the most severe form of the disease (VL); (2) cutaneous, the most common (CL); and (3) mucocutaneous. Visceral leishmaniasis is the most severe as it can become fatal if not treated and is characterized by persistent irregular fever and splenomegaly [18]. The etiologic diagnosis of HLH disease is challenging to make based on patient symptoms alone [19]. Bone marrow findings are informative in identifying amastigote forms of *Leishmania* seen within macrophages and extracellularly along with characteristic hemophagocytosis, as demonstrated in Figure 1 and Figure 3.

Leishmaniasis has regional specificity and a low incidence. The parasitological diagnosis is still the best method for diagnosing leishmaniasis due to its high specificity [16]. The amastigote forms (LD bodies) are identifiable in tissue smears obtained from the lymph nodes, bone marrow, or spleen. Following staining with Giemsa or Leishman stain, the cytoplasm exhibits a pale blue hue, while the relatively large nucleus displays a red staining. Adjacently oriented to the nucleus within the same plane lies a deep red or violet rod-like structure denoted as a kinetoplast [20,21].

Researchers found that the concurrent administration of liposomal amphotericin B (L-AmB) and miltefosine presents the potential for a novel abbreviated treatment strategy, offering the prospect of reduced miltefosine therapy duration and diminished L-AmB dosage. Furthermore, findings indicate that the sequential use of these two agents is well-tolerated and does not appear to exert a substantive adverse impact on either parasitological or clinical efficacy, as evidenced by both initial and sustained responses [21,22]. Our patient responded satisfactorily to similar treatment and showed improved blood parameters without complications. 

## Conclusions

Our case highlights an essential fact that HLH can complicate leishmaniasis, although it is a rare association. A timely diagnosis of the infectious agent causing HLH can help provide precise treatment for better outcomes, especially considering the other more common infectious agents that can trigger HLH disease, such as EBV, which requires an entirely different treatment plan. Additionally, the decision to aspirate the marrow helped visualize the parasites and active HLH, thus reaching the confirmatory diagnosis. The bone marrow aspirate findings supported the primary parasitic disease and associated HLH disease, saving critical time treating the patient. Furthermore, physicians should consider including lab testing for *Leishmania *as part of their initial approach, particularly in areas where bone marrow examination may not be readily available or after ruling out other underlying causes. 
